# Systematic and Empirical Study of the Dependence of Polyphenol Recovery from Apricot Pomace on Temperature and Solvent Concentration Levels

**DOI:** 10.1155/2018/8249184

**Published:** 2018-01-29

**Authors:** Dina Cheaib, Nada El Darra, Hiba N. Rajha, Richard G. Maroun, Nicolas Louka

**Affiliations:** ^1^Faculty of Sciences, Beirut Arab University, Riad El Solh, P.O. Box 115020, Beirut 1107 2809, Lebanon; ^2^Faculty of Health Sciences, Beirut Arab University, Tarik El Jedidah, Riad El Solh, P.O. Box 115020, Beirut 1107 2809, Lebanon; ^3^Unité de Recherche Technologies et Valorisation Agro-Alimentaire, Centre d'Analyses et de Recherche, Faculté des Sciences, Université Saint-Joseph de Beyrouth, Riad El Solh, BP 11-514, Beirut 1107 2050, Lebanon

## Abstract

This work aims to study the impact of solvent mixture (between 0 and 50% ethanol/water mixture) and temperature (between 25°C and 75°C) levels on the solid-liquid extraction of phenolic compounds (quantity and bioactivity) from apricot pomace. Results show that the mean augmentation of 1% ethanol in the range [0–12%] enhances by three times the extraction of polyphenols compared to the same augmentation in the range [0–50%]. Similarly, the mean augmentation of 1°Celcius in the range [0–25°Celcius] enhances by two times the extraction of polyphenols compared to the same augmentation in the range [0–75°Celcius]. Moreover, 1% of ethanol exhibited a greater impact on the phenolic compound extraction than 1°Celsius. The response surface methodology showed that the optimal extraction condition was reached with 50% ethanol/water at 75°C giving a total phenolic content (TPC) of 9.8 mg GAE/g DM, a flavonoids content (FC) of 8.9 mg CE/g DM, a tannin content (TC) of 4.72 mg/L, and an antiradical activity (AA) of 44%. High-performance liquid chromatography (HPLC) analysis showed that polyphenols were influenced by the selectivity of the solvent as well as the properties of each phenolic compound. Apricot pomace extracts could therefore be used as natural bioactive molecules for many industrial applications.

## 1. Introduction

The issue of food waste has always been considered of high importance because of its environmental and economic interests [1]. The use of the valuable constituents from fruit wastes is an interesting alternative for valorizing these byproducts (made up of fruit parts: seed, pulp, and skin) due to their rich content in many bioactive molecules, especially phenolic compounds [1]. In recent years, different biological activities were contributed to phenolic compounds. They act as antioxidant, anti-inflammatory, anticarcinogenic, antimicrobial and prevent many types of diseases (cardiovascular diseases, etc.) [2]. Consequently, phenolic compounds contribute to the nutritional quality of fresh and processed food and can be considered of great interest to food industry encouraging their use as natural substances or food ingredients [3]. Apricot fruit has a great nutritional value and is a rich source of beneficial bioactive compounds (vitamin C and carotenoids). It is processed on a basis of 40–45% of total world production [4]. Many studies found that some phenolic compounds (chlorogenic acid, epicatechin, and rutin) are detected in the peel and pulp of apricot in higher concentrations in the peels then the pulps [5]. Except the seed, the percentage of the pomace represents 10% of total apricot processed [6]. The extraction of bioactive molecules is considered as an important step, allowing the utilization of these compounds in many industrial products (pharmaceuticals, nutraceuticals, and cosmetics) [7]. The most widely used method of extracting polyphenols is based on solid-liquid extraction that allows the manipulation of different parameters such as time, temperature, and solvent which can affect an extraction process. That is why optimization is necessary to obtain a high phenolic yield [8]. Moreover, waste valorization has many environmental and economic advantages for the country. In the literature, two studies were only published concerning the extraction of bioactive substances (carotenoids) from apricot pomace using supercritical (SC)-CO_2_ method [6, 9]. To our knowledge, the extraction of polyphenols from apricot pomace has not yet been documented. The major aim of this work was to valorize apricot pomace remaining during apricot industrial processes (puree and juice) by studying the effect of solvent percentage and temperature gradient on the recovery of polyphenols. First, we determined the parameters: temperature and solvent (ethanol and water) that allow the extraction of the highest phenolic yield and then we analyzed the effect of these parameters on the quality of the extracts by determining the antiradical activity.

## 2. Materials and Methods

### 2.1. Raw Materials

Apricot pomace was obtained from Conserves Modernes Chtaura (Chtaura, Bekaa, Lebanon), a Lebanese industry specialized in the production of fruits purees and jams. The pomace consists of pressed skins and pulp residues. On arrival the raw material was stored at −20°C until utilization.

### 2.2. Dry Matter Content

The dry matter content of the raw material was carried out by weighing an appropriate amount of sample and then drying it for 24 h in a ventilated oven at 105°C [10].

### 2.3. Solid-Liquid Extraction Process

The extraction process of polyphenols from apricot pomace was performed with a solid-liquid ratio of 1 : 10 (w/v). Phenolic compounds' extraction was done using two variables: temperature (25**‎**°C, 50**‎**°C, 75**‎**°C) with different-percentage mixture of solvents (water, 25% ethanol, and 50% ethanol) for 210 minutes in order to determine the maximal phenolic concentration. The extracts were then centrifuged at 5000 rpm for 10 min, filtered, and then stored at −20**‎**°C for analysis.

### 2.4. Quantification of Total Polyphenol Content (TPC) by Folin-Ciocalteu Method

Determining of the total phenolic content was done by Folin-Ciocalteu method. 0.2 mL of the extract, 0.1 mL of FC reagent, and 0.8 mL of sodium carbonate solution Na_2_CO_3_ (75 mg/L) were mixed and kept for 10 min at 60°C [11]. The absorbance was measured at 750 nm by a spectrophotometer UV-VIS (Gold S54T UV-VIS, China). The measurements were compared to a standard curve of prepared gallic acid solution and expressed in milligrams of gallic acid equivalent (GAE) per gram of dry matter (mg GAE/g DM).

### 2.5. Effective Diffusivity of Solutes

Using Fick's second law, solutes diffusivity was studied. Apricot pomace molecules were considered as spheres. The solution of Fick's second law for a well-stirred solution of limited volume was given in the following equation:

(1) $$\begin{array}{c} \frac{M_{t}}{M_{\infty }}=1-\overset{\infty }{\underset{n=1}{\sum }}\frac{6\alpha \left(\alpha +1\right)exp\left(-Dq_{n}^{2}t/a^{2}\right)}{9+9\alpha +q_{n}^{2}\alpha^{2}}, \end{array}$$

where *M*_*t*_ is the polyphenol concentration in the extract after time *t*, *M*_*∞*_ is the maximal concentration of polyphenol after an infinite time, *α* is the solid/liquid ratio, *D* is the coefficient of diffusion (m^2^/s), *a* is the radius of the sample, *n* = 5, *q*_1_ = 2.4048, *q*_2_ = 5.5201, *q*_3_ = 8.6537, *q*_4_ = 11.7915, and *q*_5_ = 14.9309 [12].

### 2.6. The Effect of [1°C] and [1%] on the Extraction of Polyphenols

*[1* °*C]* represents the concentration of polyphenols extracted by the first degree Celsius. It was calculated by dividing each concentration of polyphenols by its temperature gradient (25**‎**°C, 37°C, 50**‎**°C, 62°C, or 75**‎**°C)

*[1%] *represents the concentration of polyphenols extracted by the first percentage of ethanol distributed equally for each ethanol percentage (12%, 25%, 37%, or 50%). It was calculated by dividing each concentration of polyphenols by its ethanol percentage (12%, 25%, 37%, and 50%).

### 2.7. Determination of the Antiradical Activity (AA)

The antiradical activity was examined by the DPPH (1,1-diphenyl-2-picrylhydrazyl) scavenging method. 4 mL of 1 mM DPPH (in 80% methanol) was mixed with 0.2 mL of the extracts and then left at room temperature for 30 min. The reduction of the DPPH free radical was measured by reading the absorbance at 517 nm. Methanol was used as a blank. Two models were represented: the percentage of inhibition at the initial concentration of the extracts and the inhibition percentage at a fixed polyphenol concentration (2 mg GAE/g DM) in order to screen the influence of their diversity [13]. The inhibition percentage of the DPPH free radical was calculated as follows:

(2) %  inhibition=absorbance  of  control−absorbance  of  test  sampleabsorbance  of  control∗100.

### 2.8. Determination of Tannin Content (TC)

Tannin content was determined according to Ribéreau-Gayon et al. Two tubes were prepared; each one contains 1 mL of the extract, 0.5 mL of water, and 1.5 mL of HCL (12 N). The first tube was heated at 100°C for 30 min and the second is kept at room temperature for the same duration. After rapid cooling of the tubes, 0.25 mL of ethanol was added to each one of them. The absorbance was measured at 520 nm [14]. The tannin concentration was calculated as follows:

(3) Tannin  concentrationmg/L=19.33×Δ optical  densities.

### 2.9. Determination of Total Flavonoids (TF)

Briefly, 1 mL of apricot extract was added to 4 mL of water. After 5 min, 0.3 mL of NaNO_2_ (5%) and 1.5 mL of AlCl_3_ (2%) were added. 2 mL of NaOH (1 M) was then added to the mixture after 5 min. The absorbance was measured at 510 nm. The results were expressed according to the calibration curve of catechin and the total flavonoids (TF) were expressed as mg of catechin equivalent (CE) per g of dry matter [15].

### 2.10. Experimental Design

The optimization of phenolic compounds from apricot pomace by solid-liquid extraction was carried out using response surface methodology (RSM). A design defined by the experimenter was conducted to evaluate the effect of two factors in 25 runs. The influence of the temperature (*T*) and ethanol concentration (EC) on the extraction of polyphenols, flavonoids, and tannins content from apricot pomace, as well as on the antiradical activity of the extracts, were studied. Temperature values varied between 25°C and 75°C and ethanol concentration between 0% and 50%. The time was fixed to 90 minutes. Considering two factors and four responses, experimental data were established to obtain a second-degree regression equation of the form

(4) $$\begin{array}{c} Y=\beta_{0}+\beta_{1}EC+\beta_{2}T+\beta_{11}EC^{2}+\beta_{12}EC\cdot T+\beta_{22}T^{2}, \end{array}$$

where *Y* is the predicted response parameter, EC is the ethanol concentration, *T* is the temperature, *β*_0_ is the mean value of response at the central point of the experiment, *β*_1_ and *β*_2_ are the linear coefficients, *β*_11_ and *β*_22_ are the quadratic coefficients, and *β*_12_ represents the interaction coefficient. Experimental design and statistical treatment of the results were determined using STATGRAPHICS* Plus *4.0 for Windows.

### 2.11. Experimental Points

Experimental points were chosen according to a systematic variation of temperature for the values of 25, 37, 50, 62, and 75°C and ethanol concentration for the values of 0, 12, 25, 37, and 50% covering all the possible combinations between the levels of the two variables (Table 1).

### 2.12. HPLC-DAD Analysis

Polyphenol analyses of the extracts from apricot pomace were performed by high-performance liquid chromatography (HPLC) Jasco HPLC system (PV-2089). Equipment consists of an autosampler, a Jetstream column oven, an L-2130 pump, and an L-2450 diode array detector. The separation of polyphenols was examined through a C18 column (25 × 0.46 mm). The mobile phase consisted of acidified nanopure water at pH 2.3 with HCl (A) and acetonitrile (B) HPLC grade. The elution program was done under isocratic conditions from 0 to 5 min with (85%) A and (15%) B. Gradient profile was from 5 to 30 min, beginning with (85%) A and (15%) B and ending with (0%) A and (100%) B. It was followed by isocratic conditions from 30 to 35 min with (0%) A and (100%) B, to reequilibrate the column. The injection volume was 10 *μ*L and the flow rate was 1 mL/min. The standards that were used for identification and quantification are transcinnamic acid, caffeic acid, epicatechin, chlorogenic acid, catechin, rutin, gallic acid, and kaempferol. All peaks were quantified and identified based on the retention time and the spectra of external standards of each phenolic compound. The concentration of polyphenols was determined from standard curves constructed for individual compounds by injecting different concentrations of the corresponding standards [16].

## 3. Results and Discussion

### 3.1. Kinetic Model for Polyphenols Extraction

**Figure 1:**
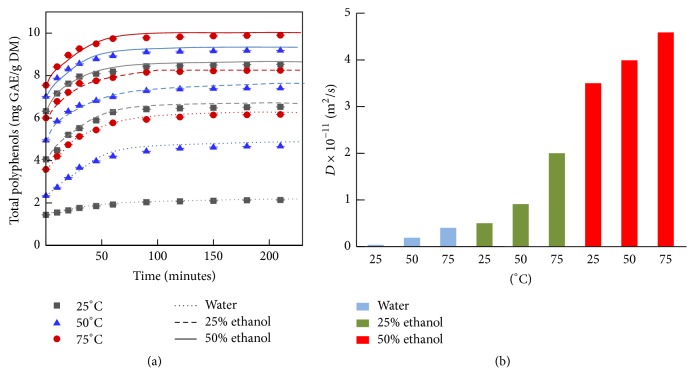
(a) Kinetics of the solid-liquid extraction of polyphenols from apricot pomace with different temperatures (25°C, 50°C, and 75°C) and ethanol concentrations (water, 25% and 50% ethanol) for 210 minutes. (b) Polyphenol diffusion coefficients of apricot pomace during the kinetics with different temperatures (25°C, 50°C, and 75°C) and solvent ratios (water, 25% and 50% ethanol).

Figure 1(a) represents the kinetic model for the solid-liquid extraction of polyphenols from apricot pomace during 210 minutes. Two variables were studied: temperature (25°C, 50°C, and 75°C) and solvents mixture (water, 25% and 50% ethanol). The phenolic yield reached its maximum after 90 minutes and remained stable until 210 minutes. This time (90 minutes) was chosen to perform the rest of the analysis in this study. The total phenolic content (TPC) ranged from 2 mg GAE/g DM for 25°C in water to 9.8 mg GAE/g DM for 50% ethanol at 75°C, which showed an improvement of 5 times of the phenolic extraction yield.

Moreover, the use of ethanol as solvent enhanced polyphenol extraction compared to water solvent. For example at *t* = 50 min, the TPC in 25% ethanol at 75°C (7.8 mg GAE/g DM) was higher than in water extraction at 75°C (5.6 mg GAE/g DM).

The transport of molecules from one part of a system to another caused by random particle motions is called diffusion [12]. The diffusion coefficients “*D*” of polyphenol are shown in Figure 1(b). Polyphenol diffusion in the extraction medium increased with the augmentation of the temperature and the percentage of ethanol/water in the solvent. With the elevation of temperature in water solution, the diffusion coefficient increased from 3.5 × 10^−13^ (for water at 25°C) to 4 × 10^−12^ m^2^/s (for water at 75°C). As for ethanol addition at different temperatures, the diffusion coefficient increased to reach a maximum of 4.59 × 10^−11^ m^2^/s for 50% ethanol at 75°C. Ethanol was shown to play an important role in polyphenol solubility and diffusion, since the capacity of diffusion depends on the solute diffusion, solvent diffusion, and solute solubility [12, 17]. Our results exhibited a significant correlation between the polyphenol diffusion and their extraction yield.

### 3.2. Polyphenol Recovery as Function of Temperature and Ethanol Percentage

**Figure 2:**
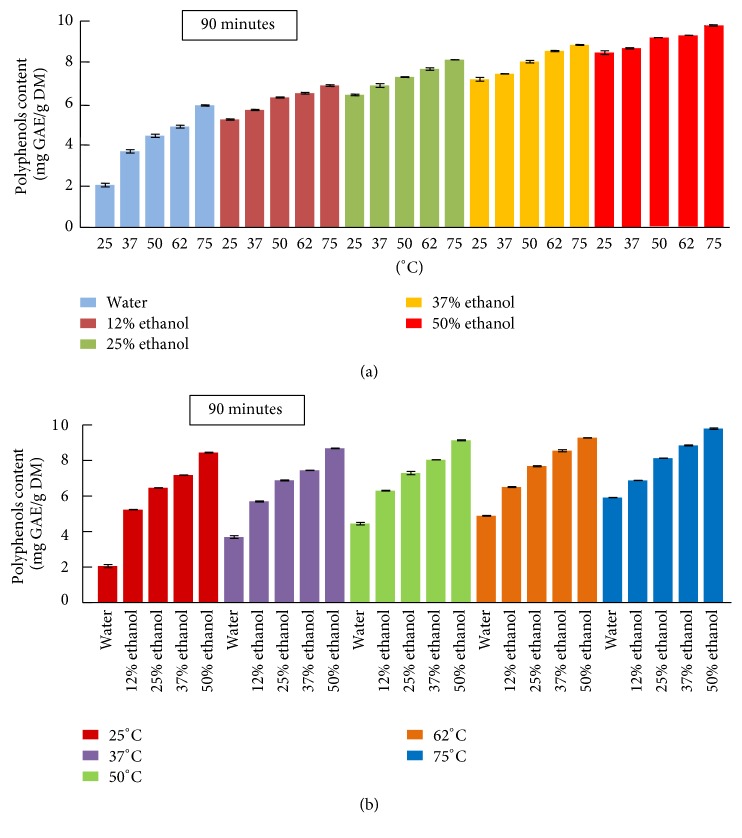
Polyphenol recovery as function of (a) temperature and (b) ethanol percentage at 90 minutes.

The comparison of polyphenol recovery as function of temperature and the ethanol percentage after 90 minutes of extraction was shown in Figures 2(a) and 2(b), respectively. The efficiency of the extraction is affected by temperature elevation as shown in Figure 2(a). This was also proven by the study of Rajha et al. (2014) which showed an enhancement in polyphenol recovery from grape pomace with the temperature elevation [18]. This effect of the temperature may be due to the increase in mass transfer, enhancement of the solubility of the solute in the solvent, and the decrease in the solvent viscosity and surface tension [19, 20]. Moreover, the efficiency of the extraction is also affected by ethanol addition (Figure 2(b)). Ethanol solvent gave higher yields of phenolic compounds than those obtained from water extraction at the same temperature. For example, the TPC in 50% ethanol at 50°C (9 mg GAE/g DM) was higher compared to water extraction at 75°C (4.4 mg GAE/g DM). This difference may be due to the high diversity of polyphenols; some are water-soluble (anthocyanins, proanthocyanidins, etc.), while others are ethanol-soluble (epicatechin, catechin, etc.) [21]. The improvement of polyphenols extraction by ethanol addition can be related to the chemical and biophysical cell membrane alteration. Ethanol influences the cell permeability by changing the phospholipid bilayer of the cell membranes [22].

### 3.3. Effect of [1°C] and [1%] on the Phenolic Compounds Extraction

**Figure 3:**
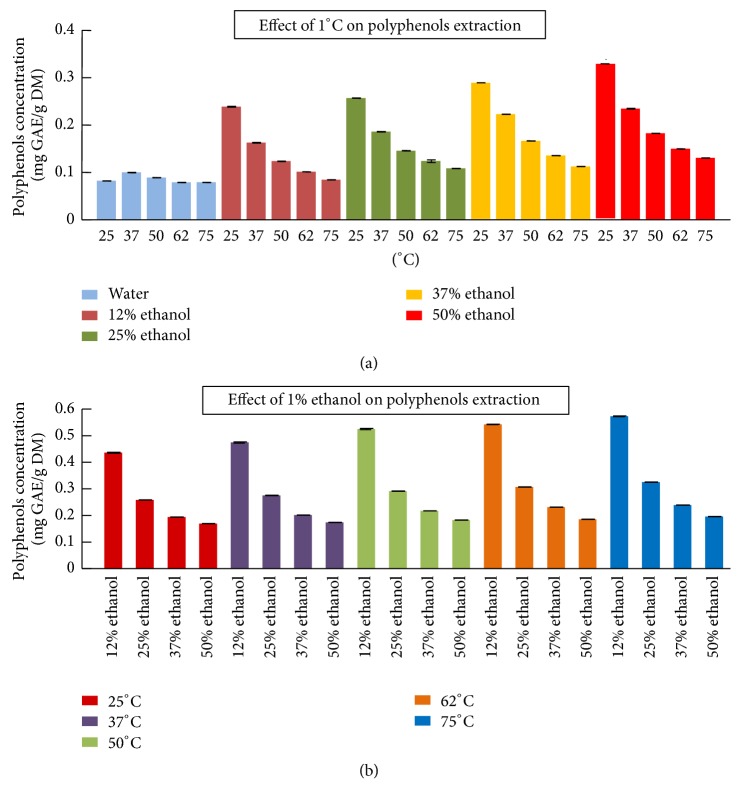
The effect of (a) 1°Celsius and (b) 1% ethanol on the phenolic compounds extraction.

Figure 3 represents the effect of (a) 1°Celsius and (b) 1% ethanol on the phenolic compounds extraction. [1°C] during water extraction did not affect the concentration of polyphenols extracted with the increase in temperature passing from 25°C to 75°C. However, with the same ethanol/water ratio, the first [1°C] had 2 times more efficiency at 25°C than at 75°C on the extraction of polyphenols. The same tendency (two times) was observed for other ethanol/water ratios (25%, 37%, and 50% ethanol). For example, [1°C] was able to extract 0.20 mg GAE/g DM (with 12% ethanol at 25°C) and 0.09 mg GAE/g DM (with 12% ethanol 75°C) (Figure 3(a)). With the increase in temperature, the effect of 1°Celsius decreased gradually to attain the same effect during water extraction. This could be explained by the fact that at low temperatures the efficacy of both factors is greater than at high ones. Regarding ethanol, the effect of [1%] at 12% ethanol was 3 times higher compared to 50% ethanol at the same temperature (Figure 3(b)). For example, 1% ethanol was able to extract 0.57 mg GAE/g DM (with 12% ethanol at 75°C) and 0.19 mg GAE/g DM (with 50% ethanol 75°C) (Figure 3(b)).

Our findings showed that [1%] of ethanol had a greater impact on the phenolic compound extraction than [1°C]. For example, 1% ethanol extracted 0.43 mg GAE/g DM while 1°C extracted 0.23 mg GAE/g DM with 12% ethanol/water at 25°C. The combination of these two factors (temperature and ethanol) played an important role in enhancing the extraction comparable to one factor alone (aqueous extraction).

### 3.4. Flavonoids and Tannins Recovery as Function of Temperature and Ethanol Percentage

**Figure 4:**
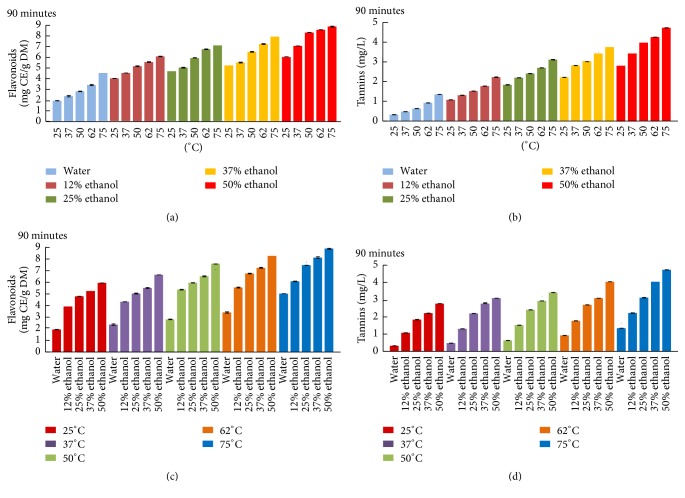
Flavonoids (a, c) and tannins (b, d) recovery of apricot pomace extracts at 90 minutes.

Figure 4 represents flavonoids (a, c) and tannins (b, d) recovery of apricot pomace by solid-liquid extraction. It is known that the yield of polyphenols extraction depends on the type of solvents, temperature, and sample-to-solvent ratio. Moreover, the solubility of the phenolic compounds is driven by the chemical nature of the fruit sample and the polarity of the solvent used [23]. Temperature elevation and the increase in ethanol percentage had positive effect on flavonoids content (Figures 4(a) and 4(c)) which attained its maximum of 8.9 mg CE/g DM with 50% ethanol/water at 75°C (which were the highest temperature and solvent ratio tested). Flavonoids are proven to be extracted efficiently from fruit by ethanol [24]. Our results are in agreement with the study of Rajha et al. (2014), which showed the effect of temperature elevation on flavonoids extraction, from grape pomace [25]. Regarding tannin content (TC), it increased gradually with temperature in water extraction to attain 1.4 mg/L at 75°C (Figure 4(b)). The efficiency of tannin extraction was enhanced by 3 times with 50% ethanol at 75°C until it reached a maximum of 4.72 mg/L (Figure 4(d)). It was demonstrated that ethanol was effective for the extraction of tannin from plant materials [26]. Rajha et al. (2012) found that the highest ethanol/water ratio tested (64% ethanol) was the most appropriate ratio for the extraction of tannins in grape pomace [27]. In concordance with total polyphenol extraction, tannins and flavonoids recovery from apricot pomace was also enhanced by both temperature and ethanol addition.

### 3.5. Radical Scavenging Effect Assay

**Figure 5:**
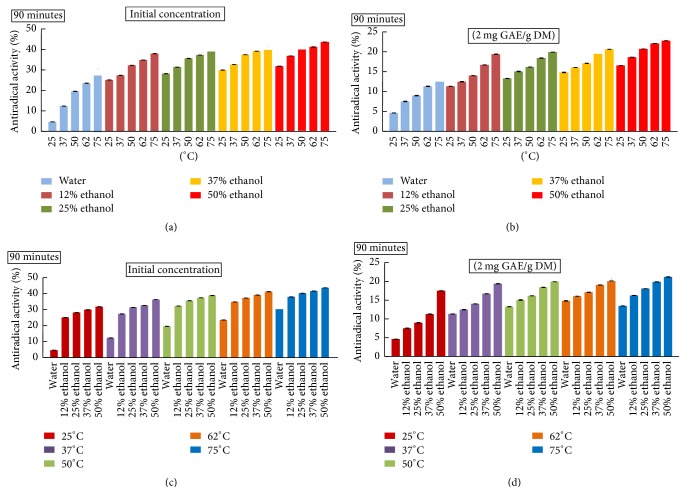
Antiradical scavenging capacity of apricot pomace (a, c) at their initial concentration and (b, d) at 2 mg GAE/g DM of polyphenols.

Figure 5 shows the antiradical activity (AA) of apricot extracts at their initial concentrations (Figures 5(a) and 5(c)) and at 2 mg GAE/g DM (Figures 5(b) and 5(d)) for different temperature and ethanol concentration. Both factors had positive effect on the AA and the highest value (44%) was obtained with 50% ethanol at 75°C corresponding to the extract presenting the highest TPC. On the other hand, many authors showed the concentration-dependent antiradical activity of phenolic compounds [25]. The same tendency was observed at the same polyphenol concentration (2 mg GAE/g DM) (Figures 5(b) and 5(d)). The increase in temperature enhanced the AA. The latter was also improved with ethanol addition (Figures 5(c) and 5(d)). This could be due to the increase in the polarity of the mixed solvent caused by ethanol which contributes to a high extraction of phenolic compounds with higher bioactivity [25].

### 3.6. Results Using the Experimental Design: Response Surface Methodology (RSM)

#### 3.6.1. Experimental Design

Response surface methodology (RSM) was conducted to determine the adequate temperature and ethanol concentration for the optimization of polyphenols, flavonoids, tannins, and the antiradical activity of extracts from apricot pomace. The optimal multiple responses were obtained with an ethanol concentration of 50% and a temperature of 75°C giving a TPC (9.8 mg GAE/g DM), FC (8.9 mg CE/g DM), TC (4.72 mg/L), and AA (44%).

#### 3.6.2. Experimental Modelization and Statistics

The regression equations allowed the calculation of the predicted values (data not shown). The coefficients of regression *R*^2^ were calculated by the analysis of the predicted value obtained by the regression models. *R*^2^ can reveal the significance of each experimental factor. The models exhibited high levels of adequacy, which are shown by the closeness to 1 of the *R*^2^ values indicating strong correlation between the observed and predicted values (Table 2). This implies that a good agreement of the corresponding model with the experimental results is found.

#### 3.6.3. Parameters Significance

**Figure 6:**
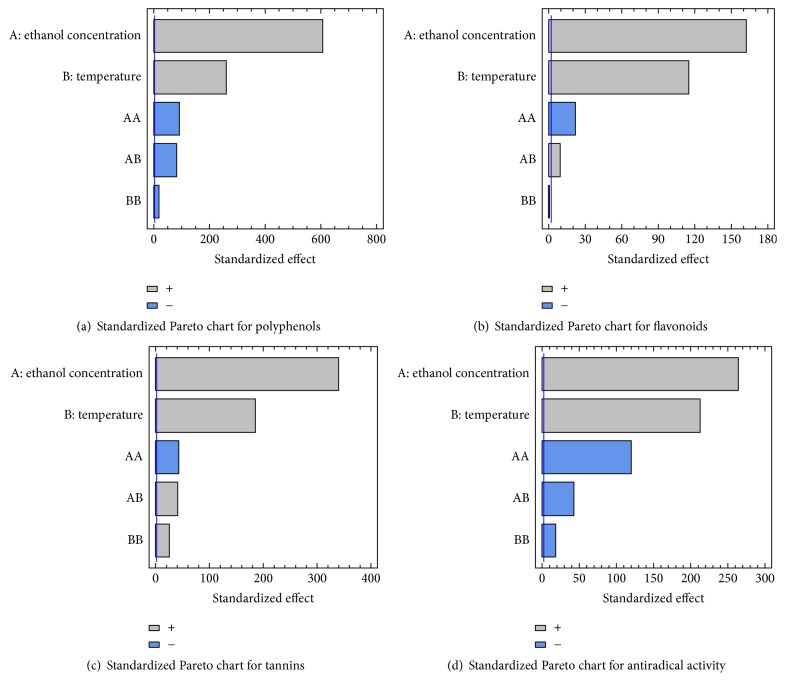
Standardized Pareto chart. Analysis shown for polyphenols (a), flavonoids content (b), tannins content (c), and antiradical activity (d). The variables are temperature and ethanol concentration. It shows the columns/parameters crossing the vertical blue line, which are statistically significant with more than 95% of confidence.

The effect of both variables (temperature and ethanol concentration) and their interactions are represented in Pareto charts. When the histograms which represent each variable cross the vertical line, they are considered as significant. According to Figure 6(a), polyphenol content is positively affected by both the temperature and ethanol concentration but negatively influenced by the quadratic effect of temperature (*T*^2^), ethanol concentration (EC^2^), and the interaction of both parameters (*T* · EC). As shown in Figure 6(b), temperature (*T*) and ethanol concentration (EC) have a significant positive effect on FC. The interaction (*T* · EC) is also positive. On the other hand, the quadratic effect of ethanol concentration (EC^2^) is negative with a nonsignificant effect of the quadratic temperature (*T*^2^) on FC. Similarly, tannin content (Figure 6(c)) is positively affected by both factors (*T* and EC) and their interaction (*T* · EC); the quadratic effect of temperature (*T*^2^) is also positive. However, the quadratic effect of ethanol concentration (EC^2^) is negative. The Pareto chart of AA (Figure 6(d)) shows that temperature and ethanol concentration affect positively the antiradical activity but are negatively affected by the quadratic effect of temperature (*T*^2^), ethanol concentration (EC^2^), and the interaction between both factors (*T* · EC) (Figure 6(d)). According to the Pareto charts, temperature and ethanol concentration have significant influence on the polyphenols, flavonoids, and tannin content, as well as on the antiradical activity more than temperature. The efficiency of the extraction was proven to be affected by many parameters (such as temperature, solvent mixture, and many others) and can be either independent or interactive [20, 28, 29].

#### 3.6.4. Effect of Temperature and Ethanol Concentration on the Extraction Treatment

**Figure 7:**
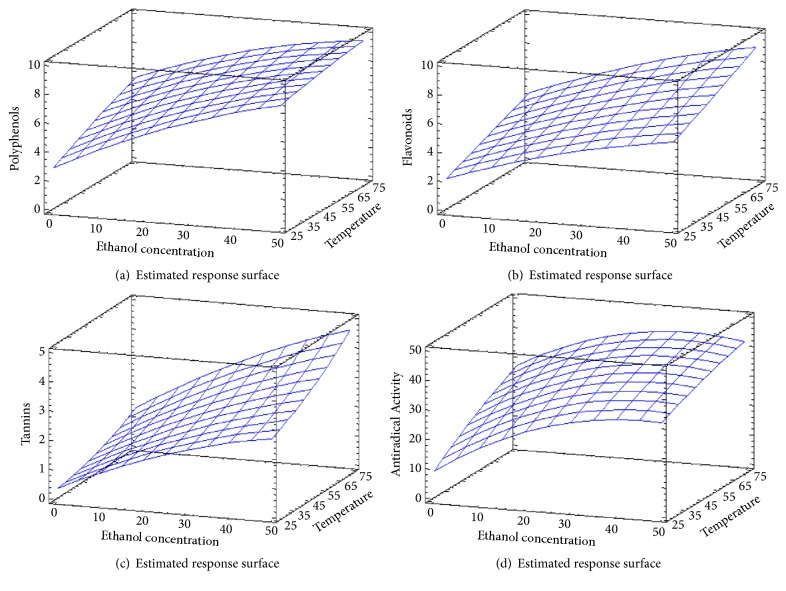
Polyphenols (a), flavonoids content (b), tannins content (c), and antiradical activity (d) surface plots. The three-dimensional graphs were plotted between two independent variables (temperature and ethanol concentration).

Besides Pareto charts, the effect of each parameter on TPC, FC, TC, and AA is represented by three-dimensional graphs. It can be noticed from Figure 7(a) that temperature and ethanol concentration have a positive linear effect on TPC since they increase with the elevation of temperature and ethanol percentage to attain an optimum of 75°C and 50% ethanol, respectively. Many studies showed that polyphenols have been efficiently extracted from different matrices with 50% ethanol. Shi et al. (2003), Seo et al. (2014), and Brahmi et al. [30] found that the highest phenolic content was reached with 50% ethanol from grape seeds, guava leaves, and Algerian mint, respectively [29, 31]. Identically to TPC, temperature and ethanol concentration have a positive linear effect on FC (Figure 7(b)). FC reached an optimum with 50% ethanol and 75°C. Temperature and ethanol percentage were demonstrated to affect polyphenols and flavonoids extraction [27, 32, 33]. Ethanol concentration has been proven to influence the phenolic compounds extraction because it reduces the boiling point and affects the polarity of the mixed solvent [34]. Temperature elevation stimulates the movement of the molecules in the sample matrix which affect the extraction of flavonoids [32]. As shown in Figure 7(c), temperature and ethanol concentration appeared to affect the concentration of tannin positively since ascent steepness reflects the increase of TC with the elevation of both factors (temperature and ethanol concentration).

TC reached its optimal value with 50% ethanol and 75°C. Ethanol which is an organic polar solvent was shown to be the most suitable solvent for the extraction of tannin. This can be explained by the fact that the polarity of ethanol enables it to have strong interactions with polar compounds such as tannin [26]. Concerning the antiradical activity represented by the inhibition percentage (Figure 7(d)), it increased with temperature elevation to attain an optimum at 75°C. Temperature shows a positive linear effect on AA. Temperature increases the coefficient of diffusion and the solubility of the solvent [35–37]. Ethanol concentration has quadratic negative effect on the AA and the optimal value for the maximization of the response was with 50% ethanol. Ethanol has been shown to enhance the extraction of phenolic compounds especially flavonoids associated with high biological properties [29, 38].

### 3.7. Quantification of Polyphenol Extracts by High-Performance Liquid Chromatography

**Figure 8:**
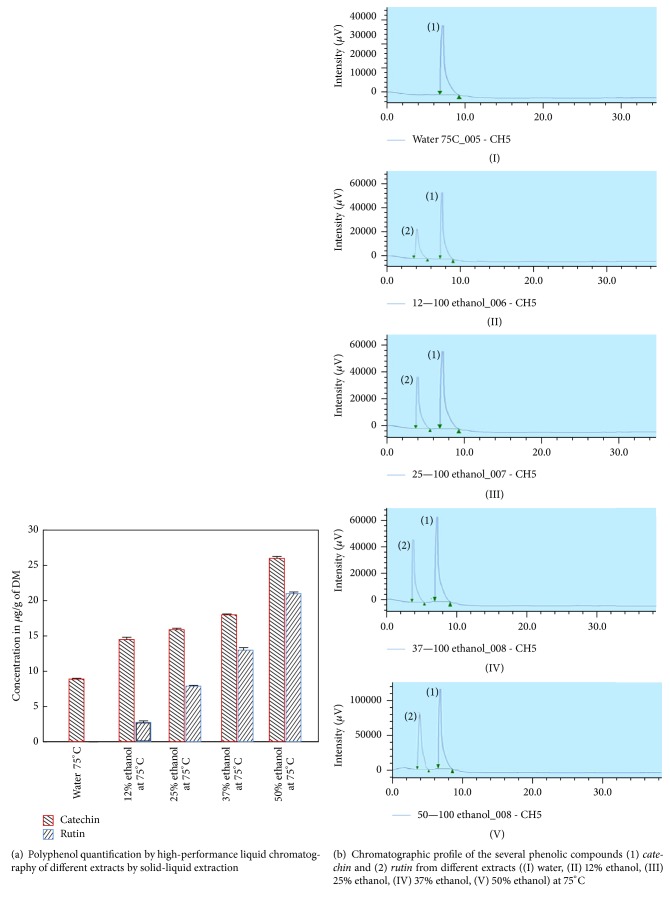


The diversity and quantity of polyphenol in different apricot extracts determined by HPLC were shown in Figure 8(a). The chromatographic profile of the several phenolic compounds from different extracts was shown in Figure 8(b). The main phenolic compounds identified in apricot pomace were catechin and rutin. These results were consistent with the results of Veberic and Stampar (2005) on the polyphenol composition in apricot varieties [39]. Polyphenols content is highly influenced by the types of solvent and the properties of the phenolic molecules of each type of fruit [40]. Moreover, alcoholic solvent (such as ethanol) is selective for some phenolic molecules, which could explain the results shown in Figure 8 [41]. Catechin is found in all samples. It is soluble in water and in polar organic solvent such as ethanol. As shown in Figure 8, catechin content increased with the elevation of ethanol percentage in the mixture. Catechin is preferentially extracted in high ethanol/water mixture [29]. Perva-Uzunalić et al. (2006) showed that water extraction of catechin from tea resulted in lower extraction efficiency in comparison to ethanol/water solvent mixture [42]. Regarding rutin, it is efficiently extracted in ethanol, which could explain its presence in all ethanol/water mixtures, and its content increased with ethanol addition [43]. Zhang et al. (2013) reported that the efficiency of rutin extraction is enhanced by the increase in ethanol percentage in the solvent from plant material [44].

## 4. Conclusion

The main objective of this work was to study the effect of ethanol percentage and temperature gradient on the recovery of polyphenols from apricot pomace. The solid-liquid extraction showed that the effect of the first augmentation of 1% ethanol and 1°C are largely superior to the same augmentation at higher levels of these parameters. Moreover, 1% of ethanol exhibited a greater impact on the phenolic compound extraction than 1°Celsius. The response surface methodology showed that the best extraction condition was reached with 50% ethanol at 75°C giving a total phenolic content (TPC) of 9.8 mg GAE/g DM, flavonoids content (FC) of 8.9 mg CE/g DM, tannin content (TC) of 4.72 mg/L, and antiradical activity (AA) of 44%. Moreover, the increase in temperature and ethanol concentration enhanced the extraction of phenolic compounds as well as the antiradical activity of the apricot extracts. Our study demonstrates that apricot pomace byproducts are considered as an important source of bioactive molecules which could be used in food industry (as preservative, antioxidant) as well as in cosmetic and pharmaceutical applications.

## Figures and Tables

**Table 1:** The experimental points of each variable for the solid-liquid extraction of polyphenols from apricot pomace.

Run	Variables	
	Temperature (°C)	Ethanol concentration (%)
(1)	25	0
(2)	25	12
(3)	25	25
(4)	25	37
(5)	25	50
(6)	37	0
(7)	37	12
(8)	37	25
(9)	37	37
(10)	37	50
(11)	50	0
(12)	50	12
(13)	50	25
(14)	50	37
(15)	50	50
(16)	62	0
(17)	62	12
(18)	62	25
(19)	62	37
(20)	62	50
(21)	75	0
(22)	75	12
(23)	75	25
(24)	75	37
(25)	75	50

**Table 2:** Second order polynomial equations relating response variables to test variables for apricot pomace. *T* is the temperature and EC is the ethanol concentration. *R*^2^, the coefficients of regression, are shown for each equation.

Regression equations
TPC = 1.05235 + 0.175454EC + 0.0757036*T* − 0.000922654EC^2^ − 0.000711745EC*T* − 0.000183165*T*^2^ (*R*^2^ = 97.88%)
FC = 0.903618 + 0.10434EC + 0.0485053*T* − 0.00072002EC^2^ + 0.000269516EC*T* + 0.0000189965*∗T*^2^ (*R*^2^ = 95.28%)
TC = 0.356553 + 0.0598738EC − 0.00786006*∗T* − 0.000465EC^2^ + 0.000385754EC*T* + 0.000283606*T*^2^ (*R*^2^ = 98.55%)
AA = −3.45552 + 1.07978EC + 0.544727*T* − 0.010899EC^2^ − 0.00336666EC*T* − 0.00169337*T*^2^ (*R*^2^ = 94.49%)
